# Attitudes and Practices from People of a Mayan Community of Mexico, Related to Tick-Borne Diseases: Implications for the Design of Prevention Programs

**Published:** 2018-06-13

**Authors:** Karla Dzul-Rosado, Cesar Lugo-Caballero, Juan Jose Arias-Leon, Freddy Pacheco-Tucuch, Gaspar Peniche-Lara, Jorge Zavala-Castro

**Affiliations:** 1 Emerging and Re-Emerging Diseases Laboratory of Centro de Investigaciones Regionales Universidad Autonoma de Yucatan, Colonia Centro, Mexico; 2 Inter-Institutional Unit of Epidemiologic and Clinical Research, Medicine Faculty, Universidad Autonoma de Yucatan, Colonia Centro, Mexico

**Keywords:** Tick-borne diseases, Community knowledge, Prevention programs

## Abstract

**Background:**

Tick-borne diseases are caused by several pathogens whose transmission could be associated to the life conditions of communities settled in endemic areas. We aimed to determine the knowledge, attitudes, and practices related to the exposition and prevention of tick-borne diseases among people living in a typical Mayan community of Yucatan, Mexico between Dec 2012 and May 2013.

**Methods:**

A directed survey was applied to 212 (100%) householders (women and men) from Teabo, Yucatan, Mexico. Answers and field notes were recorded and analyzed with central statistics.

**Results:**

People have been bitten at least once in the community, but the majority of them consider those bites innocuous. In addition, people do not consider prevention measures, and only a few mentioned the use of some chemicals on their backyards.

**Conclusion:**

This study found little awareness among the participants regarding the importance and the transmission of these diseases even though they possess a vast knowledge regarding ticks. Therefore, educational strategies and prevention programs that include these habits for its modification are required to minimize the exposition to the vectors.

## Introduction

Tick-borne diseases (TBD) are considered emerging zoonosis due to its increasing worldwide prevalence and the establishment of a complex relationship with its reservoirs and vectors with the humans (1–3). These diseases are caused by different pathogens including *Borrelia burgdorferi*, *Anaplasma phagocytophilum*, *Babesia* spp., *Rickettsia* spp. and *Ehrlichia* spp. among others (4). The transmission of TBD is associated to ecotones, which are transition regions between ecosystems, including the agricultural fields, poultry nests, and backyards, which are in close contact with the jungle (5–6). As a consequence, the main factors associated with the acquisition of TBD includes poor hygiene conditions and the closeness with pets parasitized by infected ticks inside the house (7). In northern and southern states of Mexico, there have been found pathogens like *B. burgdorferi*, *Rickettsia* spp. and *Ehrlichia* spp. in ticks from wild and domestic animals (8–12). Therefore, the increasing contact between wildlife and domestic animals serves as an amplifying mechanism for the transmission of these diseases particularly among neglected communities (2, 3, 13).

Mayan communities of Yucatan have the optimum weather and ecological conditions to sustain the transmission cycle of *Ixodes*, *Amblyomma* and *Ripicephalus* ticks among pets, cattle and wildlife (6, 7). TBD like rickettsiosis and ehrlichiosis, are endemic pathologies of the region (9, 14–24). These studies have revealed the fragility and weakness of the surveillance programs in public health, alongside with the apparent absence of habits to prevent tick bites among people. In order for those surveillance and preventive programs to work, it is important to consider the community knowledge, perceptions, and attitudes towards the problem, achieved through community participation (community engagement) strategies. The information obtained through these strategies could be used for the design of intervention strategies based on the analysis of real scenarios and not in assumptions like typically is done (25).

In these terms, by studying people from a typical Mayan community (Teabo, Yucatán, Mexico), this work aimed to determine the knowledge, attitudes, and practices related to the importance of ticks as vectors, the risk of bites and its prevention.

## Materials and Methods

### Study area

This study was carried out in the community of Teabo, Yucatan, Mexico; located between N 20° 19’ and 20° and W 89° 11’ and 89° 20’. Teabo is bordered on the north by Mayapan-Chumayel, to the south by Tekax, to the east by Cantamayec-Tixmehuac and to the west by Mani-Akil. The district population is 6205 people distributed among 1380 households which are occupying an area of 261, 87Km^2^. There is only 1 public health center. The district has a warm subhumid zone, with summer rains. The average annual tem toperature is 26.3 °C and the average annual rainfall is 65.7mm (SNIM, 2010). The study was conducted between Dec 2012 and May 2013 and it involves only the municipality of Teabo. This community was selected for three main reasons: 1) It has not been intervened by other scientific groups, 2) The daily activities of the inhabitants in the periphery areas of the households or in the outside of town are carried on in areas which are suitable for the acquisition of ticks, 3) There have been several cases of rickettsiosis among children of the community.

### Study design

A cross-sectional, descriptive study was conducted in the community among householders that were randomly selected. A household was defined as a residential unit with one or more individuals in occupation. Multiple families residing in the same household, as well as multiple structures within the same grounds, were also considered as one household. The diffusion was made through sensitization workshops organized by the health center of this community. In these workshops, the people were informed about our interest in their knowledge and traditions regarding TBD. After the workshops, the staff explained the methodology, ethical aspects, and objectives of the study to the householders that freely accepted to participate. Trained members of the working group applied a survey through a face-to-face interview with the participants. This survey was designed and validated prior the start of the study, and consisted in 10 items with its optional answers covering four different topics: (A) Knowledge (regarding the recognition of ticks and the presence of reservoirs in their houses or backyards), B) Risk of being bitten by ticks, C) Actions after a bite, D) Prevention measures to avoid tick bites. Every item consisted of a question and four or more different answers. Due to its relevance, answers and additional comments not included in the options were recorded as field noted and categorized later according to the topics. The most representative quotes are presented as part of the collected data. As this survey contains several items for variables with unknown variance, it is not possible to calculate, a sample size for a given confidence interval (26). However, it is possible to calculate a minimal sample size for a confidence interval or 95%, and a 10% maximum probability of committing type-1 error, considering a binomial distribution for several of these variables. Using: n= z^2^ (P)(Q)/d^2^ the suggestion is a minimum of 91 households to obtain relevant results (27).

### Data analysis

The surveys were checked for coding mistakes, integrity and coherency data. The data tabulation was processed and analyzed using Excel 2007® and Graph Pad 5.01, San Diego, California.

### Ethical considerations

The Research Ethics Committee of the Hospital O’Horan (Merida, Yucatan, Mexico) approved the ethical statements of this work, as a goal of the project CIE-010-1-14 registered to Dr Karla Rossanet Dzul Rosado. Both the municipal authorities and the householders were informed about the study objectives, the methodology, the questions in the survey, their faculty to choose whether to participate or not in the study without any problem for them, the anonymity of their responses and demographic data, and that they would be informed of the results of the study after its end. After these statements, informed consents (signed or fingerprinted) were obtained according to the ethics committee statements. All the photographs and quotes presented in this work were taken under consent of the portrayed people. The staff made sure that the respondents did not have cognitive disabilities previously diagnosed. The Mayan language is the most common dialect in rural communities of Yucatan like Teabo, and not all the people in the community can speak Spanish. Moreover, not all the people are able to read and write. The staff had Mayan language interpreters and aides to overcome those situations

## Results

After the sensitization workshops, 212 householders accepted to answer the survey. To obtain data regarding their general knowledge about ticks and its medical importance, people were asked about 4 main topics: knowledge of TBD, risk of being bitten by ticks, prevention measures to avoid ticks and actions performed after the tick bite. Answers collected from surveys and field notes obtained through face-to-face interviews are described below.

### Knowledge of ticks and Tick-Borne Diseases

Initially, people were asked to enumerate by its importance three of the most widely promoted infectious diseases in our region (Dengue, Chagas disease, and influenza) alongside with TBD, based on their current knowledge. About 25% of the people considered Dengue as the most important disease, followed by Influenza (9.9%), Chagas disease (6.13%) and finally TBD (0.47%) (Table 1). Related to the general knowledge, almost all of the surveyed people answered that they can identify a tick (98.5%) (Table 1). We also took field notes regarding the typical names that they use for ticks like “peech” (singular), “sojol peech” (the ticks from the dead leaves), “mejen peech” (ticks of medium size), and other variants (Table 2). The agricultural fields, labored according to their ancient traditions are called “la milpa”, whereas the jungle areas surrounding the town are called “el monte“.

**Table 1.** General knowledge regarding ticks and its medical importance (n=212)

**Topic**	**Subtopic**	**Categories**	**Number of responses**	**Percentage**
**Knowledge of ticks**	Degree of importance of some infectious diseases	Dengue	53	25.0
		Influenza	20	9.9
		Chagas’s disease	13	6.13
		Tick-borne diseases (TBD)	2	0.47
		Do not know	124	58.5
	Can identify a tick	Yes	209	98.5
		No	3	1.5
**Risk of being bitten**	Places where people have seen ticks	Inside the house	52	24.5
		In the milpa or the monte	62	29.2
		They are unaware of ticks	98	46.3
	Season of the year when their houses have been infested	During the dry season	36	17.0
		During the rainy season	79	37.2
		The house has never been infested	97	45.8
	Places in which they have been bitten	At their houses and peridomiciliary area	13	6.1
		At the milpa or the monte	64	30.2
		Never have been bitten	135	63.7
**Actions after a tick bite**	Actions are taken when they find a wound or a tick attached to their bodies	Removal of the tick and no further action	137	63.5
		Use of home remedies	75	36.5
		Seek for health assistance	0	0
	Consequences of a tick bite	It can transmit an uncomplicated disease	62	29.7
		It can transmit a mortal disease	28	13.2
		There are no consequences	3	1.4
		Do not know	119	55.7
**Preventive actions to avoid tick bites**	Actions were taken to reduce the risk of being bitten by ticks	Use of chemical agents against ticks on their yards	14	6.6
		Use of chemical agents on their dogs	13	24.5
		People that bath their dogs on a regular basis	52	6.1
		No measures	133	62.8

**Table 2.** Representative Field notes regarding the main topics explored during face to face interviews

**Main Topic**	**Subtopic**	**Quotes from the people**
**Knowledge of ticks**	Mayan Names given to ticks	Peech (Tick), Sojol peech (Ticks living in the dead leaves), K’àak’ peech (Fire tick, red-colored ticks), Mejen peech (small size ticks), Buul tá peech (Ticks living on feces).
**Risk of being bitten**	Places where they have seen ticks	At the wall of the house, the kitchen and the poultry nests
		On animals like dogs or cattle
		At the milpa (slang Mexican for agricultural fields) and the monte (slang Mexican for jungle)
		At the firewood or corncobs brought from the milpa or the monte
		On clothes of the people who work at the milpa or the monte
	Season of the year when ticks are more abundant	October 4^th^ which is the day of St Francis for Catholicism. During this time because St Francis blesses the seeds and brings the ticks attached to his ankle.
		During the rainy season (June-October)
		During February and October
		Whenever there is heat
	Tick bites to the household members	I have been bitten on my back
		All my sons have been bitten several times
		The peech has bitten my husband several times at the milpa
		The children are bitten by ticks when they hug people who return from the milpa or the monte
**Actions after a tick bite**	Actions after the tick bite	I apply alcohol or Vaporub (a commercial ointment with alcanphor/menthol)
		I wash the wound with hot water, salt, and soap
		I apply Denate (methomil powder used to kill ants) on the wound
		I do nothing
	Consequences of the tick bite	It can kill people
		The bite is only dangerous if the tick has bitten a snake before
		It only makes you loose the appetite
		It produces “aax” (warts, in Mayan language)
		Nothing except for some itch
**Preventive actions to avoid tick bites**	Strategies to avoid infestation by ticks	I bath my dog with Asunthol (coumaphos)
		I bath my dogs with chlorine, detergent and “blue soap”
		I bath my dogs with “burned oil” (car oil already used and discarded)
		I spread insecticide on my yard
		I keep my dogs clean because they accompany me to the milpa

### Risk of being bitten

**Fig. 1.**
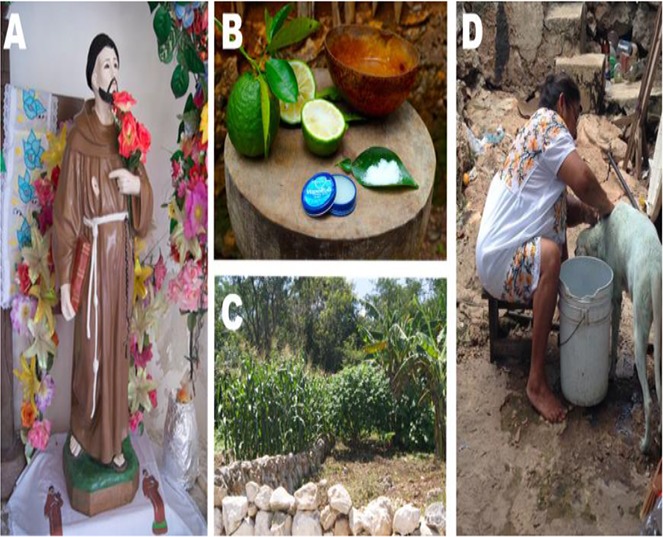
Habits and attitudes regarding ticks in Teabo, Yucatan, Mexico People

People have seen ticks inside their houses (24.5%), in “la milpa” or “el monte” (29.2%); however, 46.3% is unaware of ticks (Tables 1, 2). The term “tick infestation” was described to the interviewed people as the presence of large and noticeable quantities of ticks in the walls and floor of their houses. Following this definition, 45.8% reported not to have had tick infestations in their houses, 37.2% reported to have had infestations during rainy season (Jun–Nov), and the remaining 36% during dry season (Table 1). During the interviews, it was interesting the recurrent mention of a Catholic patron named Saint Francis, worshiped in Teabo. These people believe that “the saint has a tick attached to his ankle, so when the saint bless the milpa on Oct 4^th^ (during rainy season), the ticks fall to the ground.” (Fig. 1, Table 2). Some other people also believe that “it is written in the bible (a holy book in Catholicism) that Saint Francis protects the ticks.” (Fig. 1, Table 2). Finally, people answered that 24.5% have been bitten in the house, 29.2% in *“*la milpa*”* and 46.2% deny have been bitten (“I have been bitten on my back”, “The peech has bitten my husband at la milpa”, “All my sons have been bitten several times”) (Tables 1, 2).

### Actions after a tick bite

After the tick bite, most people do not perform further actions and just remove the tick (63.5%) because “nothing bad happens”. However, 36.5% of the surveyed people use home remedies (Tables 1, 2). These home remedies include water (I only wash the bite site with water and salt”), ointments (“I apply Vaporub to the bite”, “I apply alcohol, and that is all you should do.”) and insecticides (“I apply a mixture of “Denate” (methomyl/diflubenzuron) and water to the bite”) (Fig. 1, Table 2). Actually, 55.7% of the people ignore the consequences of a tick bite, 1.4% said that there are no consequences (“It only makes you lose your appetite”, “It provokes an aax (In the Mayan language, the word *“*aax*”* means warts)”, “Nothing, it is not dangerous. It gives you itches sometimes.”). In contrast, 42.9% knows that the bite can transmit a disease, and from these people, 13.2% knows that the disease can be mortal (Tables 1, 2). However, their comments show that the people do not know what kind of disease is (“It provokes an aax”, “Only if the tick has bitten a snake, its bite can kill you”).

### Prevention measures to avoid tick bites

Regarding the topic of prevention, 62.8% of the people do not use measures to prevent tick infestation and bites. The remaining 37.2% has some kind of practice. Within this group, 24.5% uses chemical agents on their dogs in addition to the bath (“I bathe my dogs with asuntol and a special talc powder.”), detergent (I bathe my dog with fab and blue soap.”) or other kind of chemicals (“I bathe my dogs with burned oil.”, “I bathe my dogs with chlorine and blue soap.”), 6.1% just bathes their dogs with preventive soaps, 6.6% uses any chemical agent against ticks on their yards (“I spread insecticide in my yard”) (Fig. 1, Tables 1, 2). For the people in Yucatan, asuntol is the common name of several brands of ectoparasiticides containing Coumaphos, and fab is the generic name for several brands of detergents.

## Discussion

Rural towns in Yucatan meet the ecological and environmental conditions to sustain the life cycles of several infectious disease vectors including ticks. Therefore, many vector-borne diseases including TBDs like rickettsiosis and ehrlichiosis are considered endemic pathologies in Yucatan (9, 15–24). As with other zoonotic diseases, there must be two main objectives to prevent the transmission of TBDs. The first one is to achieve a change in the habits of people (keep the animals clean, search for ticks after a hike through the jungle among others), and the second one involves the improvement of the peridomiciliary area (removal of trash and leaves, keep down the grass and vegetation as an example). Both goals could be achieved through community intervention programs (28). When a population at risk to acquire a TBD has been sensitized after these types of strategies, the level of awareness could be gradually increased (29, 30). In these terms, the aim of this work was to inquire the knowledge and habits of a community under risk to acquire TBDs, through face-to-face directed interviews with people of a Mayan community.

### Knowledge of ticks and risk of being bitten

Less than 0.1% of the participants consider TBD among the important infectious diseases reflecting the lack of government programs against these diseases. Considering that Yucatan is an endemic area for TBD, there is a real need to design such programs, being the acquisition of knowledge the first step to achieve this goal. Ticks have several Mayan names, related to its distribution or to its characteristics like size or color. The collected data and the comments of the people suggest that they are used to the presence of ticks, which has to lead them to think that “ticks are not a threat”. It was surprising to found that the patron saint of Teabo is associated to ticks, and some people consider Oct 4^th^ (Saint Francis day at the beginning of fall) as the day when ticks spread within the town. Several scientific kinds of literature have reported than ticks, tend to be more abundant on dry rather than rainy season and that there is a dramatic reduction in its activity starting on fall (31–33). In contrast with scientific reports, the surveys did not show a clear tendency within the community, to consider than ticks are more abundant on rainy (37.3%) or dry season (36%). This data suggests that people from Teabo are not aware of the behavior of ticks even when they associate a particular date (October 4^th^) with its “sudden increase”. As the harvest season starts in Oct, this “increase” could be related to a closer contact with high grass, bushes, and crops, which puts them in a riskier situation to acquire ticks. However, as there is no information about seasonality differences on distribution of ticks in our region, this is an interesting topic to explore in depth.

### Actions after a tick bite

Some studies have reported an overestimation of the infected tick percentages leading to a saturation of health care centers by asymptomatic bitten patients (1) however; the people from this study tend to not perform further actions because their knowledge about the risks associated to these bites is scarce. Even the people that mentioned the appearance of a wound on the bite site discarded the health care center as an option to seek guidance. Similar to studies in other endemic countries, people who are not aware of this risk, only seek health care when a relative is ill or when they have the money to cover the cost of a treatment (13). Instead of this, people from Teabo use home remedies like ointments, different mixes of sour orange and salt, among others. These remedies are commonly used even then its activity against ticks has not been evaluated. There are reports of ethno practices successfully used against ticks based on the exploitation of local natural resources (34, 35). The implementation of this kind of measures alongside with the determination of its utility is an interesting topic to explore in depth.

### Prevention actions to avoid tick bites

The 62.8% of the people from this community do not use measures to prevent tick infestation in their houses. The remaining percentage uses chemical methods on their dogs, and only 6.6% uses any method on their yards. In contrast with other vectors like mosquitoes or bedbugs, ticks elimination is highly difficult. The preventive actions were the only way to stop the spreading of TBDs. Related to this, preventive measures like the use of protective clothing or repellents were not mentioned even when people were intentionally questioned about it, contrasting with other endemic countries whose health departments have permanent educational campaigns (1, 3, 30). People from communities at risk like Teabo do not perceive ticks as a threat but as a normal component of the environment with the sole activity of bothering their dogs or them, unaware of their potential consequences as it has been found in other endemic countries, even with permanent educational campaigns (1, 3).

### Limitations

The survey lacked questions that could have been important to obtain information considered in other studies like symptoms of specific diseases and its evolution.

## Conclusions

In rural communities like Teabo, TBDs are not considered a problem due to the lack of governmental prevention programs similar to others that exist for pathologies like dengue, and because they believe that ticks are harmless. As a consequence, people who underestimate or neglect the risk will not easily adopt preventive measures. People empower a vast knowledge regarding ticks. However, the knowledge on the importance and prevention of TBDs is scarce. To overcome this situation, data obtained should be taken into account to develop programs to sensitize and change to some extent, the habits and attitudes that promote the spread of TBD. These programs should focus on the development of training materials and maintenance workshops for the community and the health workers, as the empowerment and passage of these new conducts by the people are necessary elements to build awareness among the population regarding the importance of ticks as vectors of pathogens and the protection steps that they could use to minimize their risk.
